# Supplementary material for: Subject-independent decoding of affective states using functional near-infrared spectroscopy
Source: PLoS One. 2021 Jan 7;16(1):e0244840. doi: 10.1371/journal.pone.0244840 (PMC7790273; doi:10.1371/journal.pone.0244840)
Supplement: S1 File — (DOCX) [file pone.0244840.s001.docx]

**S1 File**

This document provides supplementary material to the manuscript “Subject-independent decoding of affective states using functional near-infrared spectroscopy” by Trambaiolli et al.

**Evaluation of mood and its correlation with classification accuracy:**

**Table S1** – Correlation between mood scores (VAMS) [1] and the classification accuracy with best performance in each block. * p-values are Bonferroni corrected for 64 multiple comparisons (2 blocks x 16 mood scores x 2 measurement points).

| **Reactive block, Negative vs Neutral classification using 40% of features** | | | | |
| --- | --- | --- | --- | --- |
|  | ***Pre experiment mood*** | | ***Delta mood*** | |
| ***Mood*** | ***Spearman's rho*** | ***p-value**** | ***Spearman's rho*** | ***p-value**** |
| Drowsiness | 0.005 | 1.000 | -0.196 | 1.000 |
| Agitation | 0.129 | 1.000 | -0.190 | 1.000 |
| Strength | 0.141 | 1.000 | -0.305 | 1.000 |
| Confusion | -0.248 | 1.000 | 0.334 | 1.000 |
| Agility | -0.412 | 1.000 | -0.226 | 1.000 |
| Apathy | 0.158 | 1.000 | -0.137 | 1.000 |
| Satisfaction | -0.034 | 1.000 | -0.073 | 1.000 |
| Worry | -0.288 | 1.000 | -0.017 | 1.000 |
| Perspicacity | -0.040 | 1.000 | -0.152 | 1.000 |
| Stress | -0.442 | 1.000 | 0.266 | 1.000 |
| Attention | -0.082 | 1.000 | -0.045 | 1.000 |
| Capacity | 0.112 | 1.000 | 0.131 | 1.000 |
| Happiness | -0.016 | 1.000 | -0.160 | 1.000 |
| Hostility | 0.104 | 1.000 | 0.114 | 1.000 |
| Interest | 0.188 | 1.000 | -0.009 | 1.000 |
| Introspection | -0.118 | 1.000 | -0.052 | 1.000 |
|  |  |  |  |  |
| **Active block, Positive vs Neutral classification using 5% of features** | | | | |
|  | ***Pre experiment mood*** | | ***Delta mood*** | |
| ***Mood*** | ***Spearman's rho*** | ***p-value**** | ***Spearman's rho*** | ***p-value**** |
| Drowsiness | 0.288 | 1.000 | -0.230 | 1.000 |
| Agitation | -0.007 | 1.000 | 0.042 | 1.000 |
| Strength | -0.374 | 1.000 | -0.177 | 1.000 |
| Confusion | -0.291 | 1.000 | 0.392 | 1.000 |
| Agility | -0.185 | 1.000 | 0.003 | 1.000 |
| Apathy | 0.118 | 1.000 | -0.067 | 1.000 |
| Satisfaction | 0.019 | 1.000 | 0.088 | 1.000 |
| Worry | -0.120 | 1.000 | 0.084 | 1.000 |
| Perspicacity | 0.204 | 1.000 | -0.029 | 1.000 |
| Stress | -0.179 | 1.000 | -0.060 | 1.000 |
| Attention | -0.150 | 1.000 | 0.245 | 1.000 |
| Capacity | -0.122 | 1.000 | 0.161 | 1.000 |
| Happiness | 0.489 | 1.000 | -0.183 | 1.000 |
| Hostility | 0.306 | 1.000 | -0.007 | 1.000 |
| Interest | -0.047 | 1.000 | 0.069 | 1.000 |
| Introspection | -0.240 | 1.000 | -0.050 | 1.000 |

**Complementary analysis with band-pass filter between 0.01 and 0.2 Hz:**

To evaluate if a wider frequency range for the band-pass filter would affect the classification accuracy, we repeated the experiment preprocessing steps presented in the Section 2.4.1 from the manuscript, but using a digitally band-pass filtered by a linear-phase FIR filter between 0.01-0.2 Hz. Other feature extraction, feature selection, classification, and statistical analyses followed the same steps described in the manuscript.

The LDA accuracy boxplots are presented in Figure S1. Classification accuracy for passive elicitation significantly exceeded chance level in “Positive vs. Negative” comparisons, with highest result as 64.50±12.44% (mean ± standard-deviation, p<0.05) using 20% of features (12 channels), and in “Negative vs. Neutral” comparisons (67.75±14.45%, p<0.05, 40% of features – 25 channels). No significant differences were found for “Positive vs. Neutral” comparisons.

During active elicitation comparisons, “Positive vs. Neutral” accuracies were greater than chance level, and the highest result was 71.00±17.95% (p<0.05) using only 5% of the features (3 channels). No significant differences were found for “Positive vs. Negative” and “Negative vs. Neutral” comparisons.


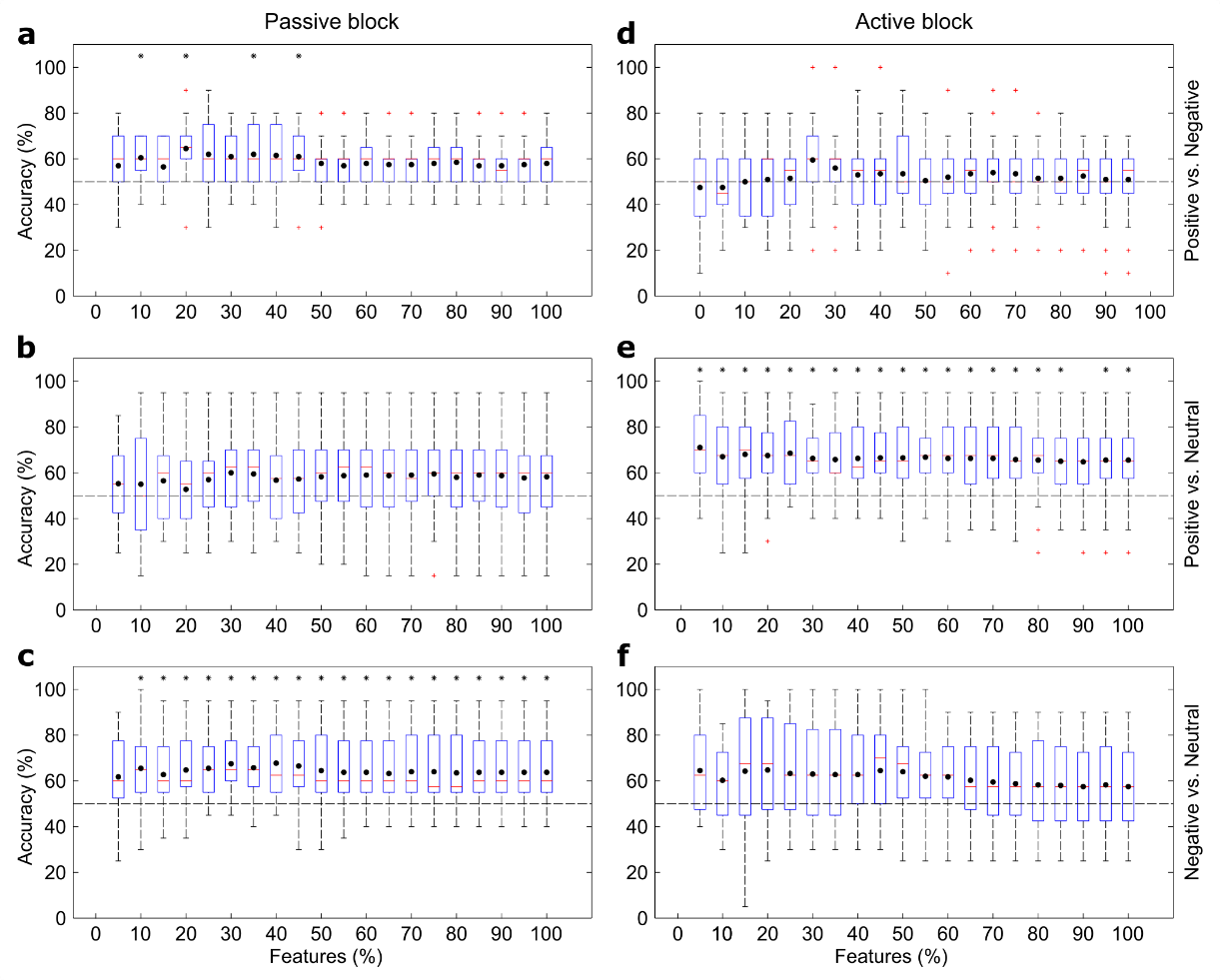


Figure S1 - Box plots showing the results using the LDA classifier and different feature subsets. (A) presents the “Positive vs. Negative”, (B) the “Positive vs. Neutral”, and (C) the “Negative vs. Neutral” comparisons for passive elicitation block, while (D-F) follows the same order for active elicitation block. Black dots present the means, red lines the medians, red crosses the outliers and black asterisks the statistical difference for chance level (p<0.05).

Compared with the results presented in the manuscript, the highest classification accuracies in each block are considerably similar (differences lower than 1%) to those achieved with a band-pass filter between 0.01 and 0.01 Hz.

**Complementary classification across datasets using Support Vector Machines:**

To evaluate if our results were dependent on the classifier, we repeated the feature selection and the classification steps using a Support Vector Machine (SVM) classifier with linear kernel and cost equal to 0.3 (default configuration from the BCILAB toolbox [2]). Feature extraction and statistical analysis followed the same steps described in the manuscript.


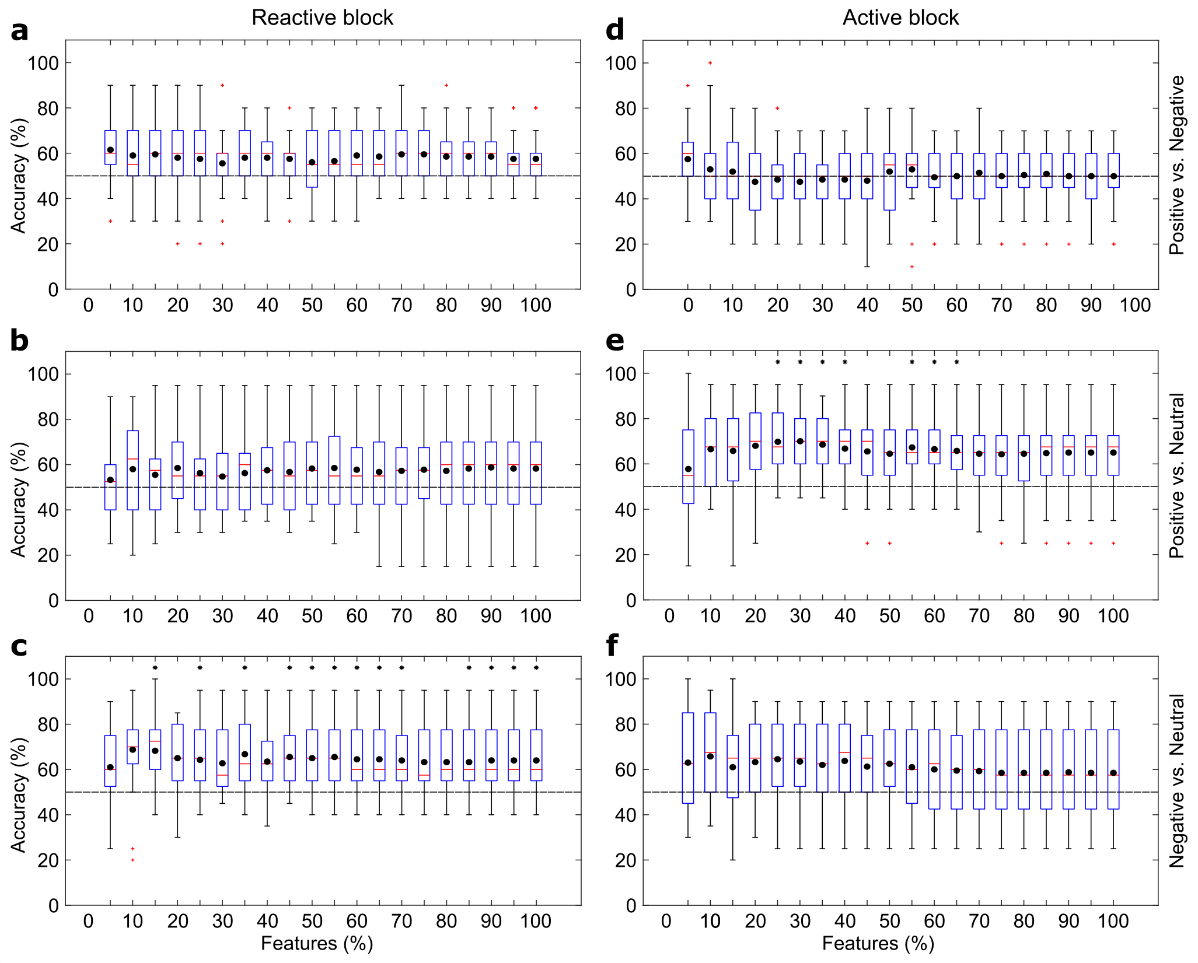


Figure S2 - Box plots showing the results using the SVM classifier and different feature subsets. (A) presents the “Positive vs. Negative”, (B) the “Positive vs. Neutral”, and (C) the “Negative vs. Neutral” comparisons for passive elicitation block, while (D-F) follows the same order for active elicitation block. Black dots present the means, red lines the medians, red crosses the outliers and black asterisks the statistical difference for chance level (p<0.05).

The SVM accuracy boxplots are presented in Figure S2. In the reactive block, significance was observed in the comparison “Negative vs. Neutral”, with 68.75±19.86% (mean ± standard-deviation, p<0.05) of accuracy using 10% of the available features (6 channels). For the active block, the “Positive vs. Neutral” comparison had 70.00±15.22% (p<0.05) as the highest performance when using 30% of features (19 channels). No significant results were observed in other comparisons in both blocks.

With exception to the absence of significance for “Positive vs. Negative” comparisons in the reactive block, the results using SVM are considerably similar to those observed using the LDA classifier (differences in accuracy lower than 3%).

**Complementary classification using leave-one-subject-out cross-validation:**

We also evaluated if the accuracies achieved using the independent samples would be sustained when using a cross-validation approach. For this, we merged data from all 69 subjects, and performed the feature selection and classification steps using a leave-one-subject-out (LOSO) cross-validation. Feature extraction and statistical analysis followed the same steps described in the manuscript.


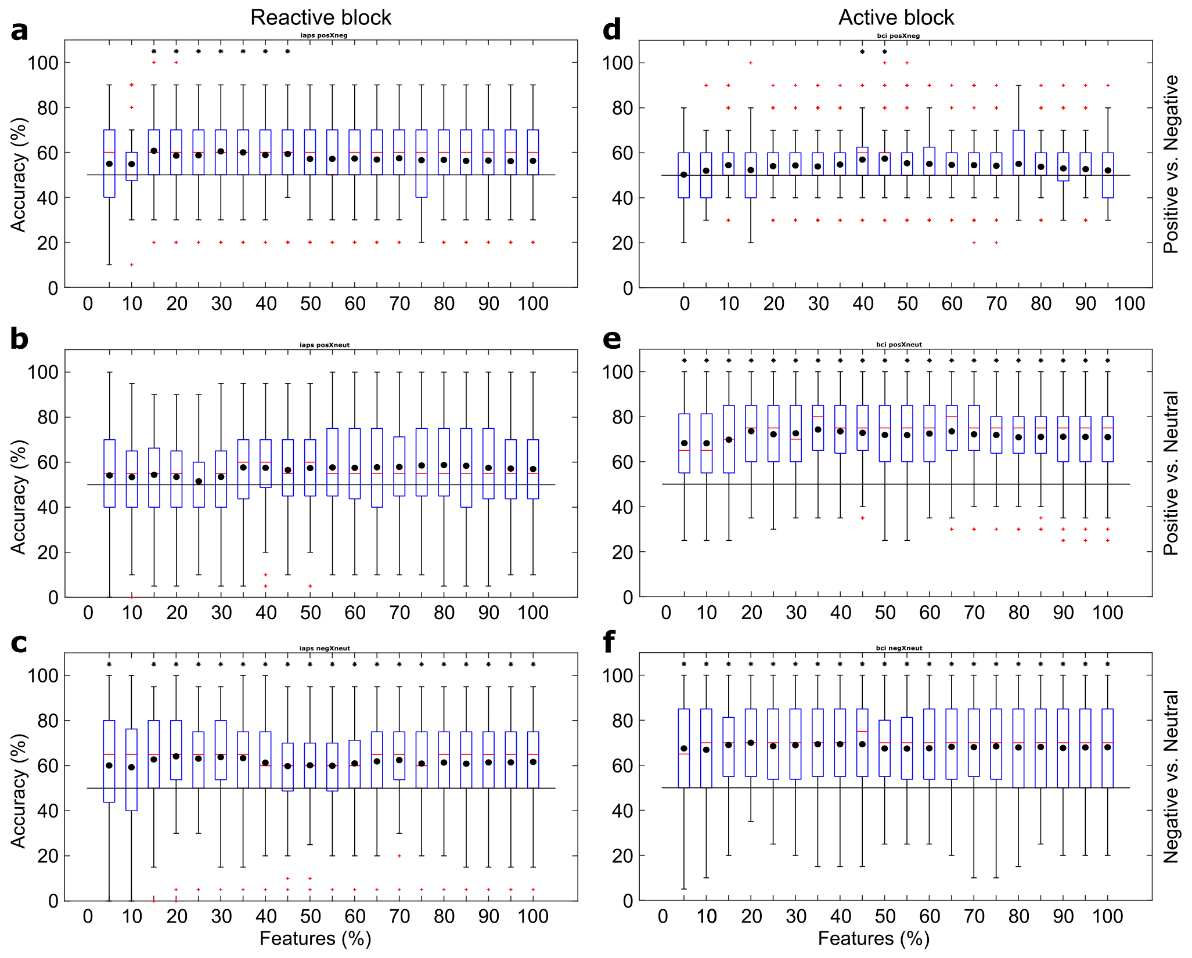


Figure S3 - Box plots showing the results using the LDA classifier and different feature subsets in a leave-one-subject-out cross-validation setup. (A) presents the “Positive vs. Negative”, (B) the “Positive vs. Neutral”, and (C) the “Negative vs. Neutral” comparisons for passive elicitation block, while (D-F) follows the same order for active elicitation block. Black dots present the means, red lines the medians, red crosses the outliers and black asterisks the statistical difference for chance level (p<0.05).

The LDA accuracies during LOSO cross-validation are shown in Figure S3. Accuracies for passive elicitation were significantly higher than the chance level in “Positive vs. Negative” comparisons, with highest result as 60.72±14.78% (mean ± standard-deviation, p<0.05) using 15% of features (9 channels), and in “Negative vs. Neutral” comparisons (64.13±20.20%, p<0.05, 20% of features – 12 channels). No significant differences were found for “Positive vs. Neutral” comparisons.

For active elicitation comparisons, “Positive vs. Negative” classification was significantly higher than change when using 50% of the features (32 channels, 57.39±13.68% and p<0.05). During “Positive vs. Neutral” comparisons the best performance was 74.28±15.65% (p<0.05), using 35% of features (22 channels), while the best accuracy during “Negative vs. Neutral” comparisons was 70.00±19.87% (p<0.05) with 20% of features (12 channels).

Compared with the results presented in the manuscript, the highest classification accuracies in each block are considerably similar (differences between 3% and 4%) to those achieved using both datasets independently. One important aspect of these results however, is that the exact null distribution in leave-one-out procedures is unknown [3]. Thus, these p-values are only approximated.

**References**

[1] R. Stern, J. Arruda, C. Hooper, G. Wolfner, and C. Morey, "Visual analogue mood scales to measure internal mood state in neurologically impaired patients: Description and initial validity evidence," *Aphasiology,* vol. 11, no. 1, pp. 59-71, 1997.

[2] C. A. Kothe and S. Makeig, "BCILAB: a platform for brain–computer interface development," *Journal of neural engineering,* vol. 10, no. 5, p. 056014, 2013.

[3] Q. Noirhomme *et al.*, "Biased binomial assessment of cross-validated estimation of classification accuracies illustrated in diagnosis predictions," *NeuroImage: Clinical,* vol. 4, pp. 687-694, 2014.
